# Association of HLA-DR1 with the allergic response to the major mugwort pollen allergen: molecular background

**DOI:** 10.1186/1471-2172-13-43

**Published:** 2012-08-08

**Authors:** Bernhard Knapp, Gottfried Fischer, Dries Van Hemelen, Ingrid Fae, Bernard Maillere, Christof Ebner, Wolfgang Schreiner, Barbara Bohle, Beatrice Jahn-Schmid

**Affiliations:** 1 Department for Biomedical Computersimulation and Bioinformatics, Medical University of Vienna, Vienna, Austria; 2 Department of Blood Group Serology, Medical University of Vienna, Vienna, Austria; 3 Department of Pathophysiology and Allergy Research, Medical University of Vienna, Vienna, Austria; 4 CEA, iBiTecS, Service d’Ingénierie Moléculaire des Protéines (SIMOPRO), Gif-sur-Yvette, France; 5 Allergieambulatorium Reumannplatz, Vienna, Austria; 6 Christian-Doppler Laboratory for Immunomodulation, Medical University of Vienna, Vienna, Austria

**Keywords:** HLA association, Peptide/MHC-class II-TCR interaction, T cell recognition, Allergen, Art v 1, Molecular dynamics simulation

## Abstract

**Background:**

Mugwort pollen allergens represent the main cause of pollinosis in late summer. The major allergen, Art v 1, contains only one single immunodominant, solely HLA-DR-restricted T cell epitope (Art v 1_25-36_). The frequency of HLA-DRB1*01 is highly increased in mugwort-allergic individuals and HLA-DR1 serves as restriction element for Art v 1_25-36_. However, Art v 1_25-36_ also binds to HLA-DR4 with high affinity and DR1-restricted Art v 1_25-36_ -specific T cell receptors can be activated by HLA-DR4 molecules. To understand the predominance of HLA-DR1 in mugwort allergy in spite of the degeneracy in HLA/peptide-binding and TCR-recognition, we investigated the molecular background of Art v 1_25-36_ /MHC/TCR interactions in the context of HLA-DR1 compared to -DR4.

**Results:**

The majority of Art v 1_25-36_ -specific T cell lines and clones from HLA-DR1 carrying, mugwort pollen-allergic donors reacted to synthetic and naturally processed Art v 1–peptides when presented by HLA-DR1 or HLA-DR4 expressing antigen presenting cells. However, at limiting peptide concentrations DR1 was more effective in T cell stimulation. In addition, the minimal epitope for 50% of Art v 1_25-36_ -specific T cells was shorter for DR1 than for DR4. *In vitro* binding assays of Art v 1_25-36_ mutant peptides to isolated DR1- and DR4-molecules indicated similar binding capacities and use of the same register. *In silico* simulation of Art v 1_25-36_ binding to HLA-DR1 and -DR4 suggested similar binding of the central part of the peptide to either molecule, but a higher flexibility of the N- and C-terminal amino acids and detachment at the C-terminus in HLA-DR1.

**Conclusions:**

The predominance of HLA-DR1 in the response to Art v 1_25-36_ may be explained by subtle conformation changes of the peptide bound to DR1 compared to DR4. Computer simulation supported our experimental data by demonstrating differences in peptide mobility within the HLA-DR complex that may influence TCR-binding. We suggest that the minor differences observed *in vitro* may be more relevant in the microenvironment *in vivo*, so that only presentation by HLA-DR1, but not -DR4 permits successful T cell activation.

## Background

CD4+ T helper (Th) cells play a major role in the induction and maintenance of IgE-mediated allergy [1], which is characterized by an excessive Th2 response. The Th2-cytokines, interleukin-4 and interleukin-13, lead to the production of allergen-specific IgE antibodies which bind to high affinity receptors on effector cells. Upon renewed allergen contact, cross-linking of these IgE antibodies leads to the release of inflammatory mediators, which cause allergic symptoms. Allergen-specific CD4+ Th cells recognize their cognate peptide via their T cell receptor (TCR) in the context of self major histocompatibility complex (MHC) class II molecules on the surface of antigen presenting cells (APC). Binding of the processed peptides to the human leukocyte antigen (HLA) class II molecules HLA-DR, -DP or –DQ occurs in endosomal compartments and is facilitated by accessory molecules. These HLA class II molecules are highly polymorphic within the population and for many immunogenic peptides degenerate binding to different MHC-class II molecules has been described. This feature is of interest for instance in vaccine development as it would allow presentation of such T cell epitopes on APC within a wide population range [2,3]. Besides, also for TCR a plasticity in ligand recognition, i.e. degeneracy in peptide reactivity as well as cross-recognition in the context of distinct MHC-molecules has been found [4,5].

In Europe and parts of Asia, mugwort (*Artemisia vulgaris*) pollen causes the most prevalent pollen allergy in late summer and autumn. Mugwort pollen contains only one major allergen, Art v 1, recognized by IgE in 95% of mugwort pollen-allergic individuals [6]. In contrast to other relevant major allergens, Art v 1 contains only one single immunodominant T cell epitope, Art v 1_25-36_ (KCIEWEKAQHGA) which is recognized by more than 80% of Art v 1-reactive mugwort pollen-allergic patients [7]. The presentation of Art v 1_25–36_ is exclusively HLA-DR-restricted and is highly associated with the HLA-DR1 phenotype [8]. Other associations between HLA class II phenotypes and allergic responses to major allergens are by far less strong [9].

Although the T cell response to Art v 1_25-36_ is clearly associated with HLA-DR1, we previously found Art v 1_25-36_–specific T cells restricted by HLA-DR3, -DR16 or -DR15 in the minor fraction of DR1-negative patients [10]. Accordingly, the peptide showed degenerate high affinity binding to several HLA-DR alleles [10]. We also observed that specific T cell activation was inducible by APC expressing DR4 or other non-DR1-HLA alleles suggesting TCR cross-recognition. Although DR4 seemed to be able to present the immunodominant Art v 1 epitope, the frequency of DR4 in our Art v 1-reactive patient group (10,7%; n = 75) is not increased compared to the normal population (16%; n = 100). Notably, we have also not been able to isolate DR4-restricted Art v 1_25-36_-specific T cells from DR1-negative, DR4-positive subjects (n = 8/75) during our intense attempts to establish TCL and TCC from mugwort-allergic patients. This discrepancy between HLA-DR4 and -DR1 is striking, as both molecules belong to the same HLA-DR supertype that includes DRB1*01:01, DRB5*01:01, DRB1*15:01, DRB1*04:01, DRB1*07:01, DRB1*09:01, and DRB1*13:02 which all show overlapping peptide-binding repertoires [11]. It is unlikely that the uniform T cell response to Art v 1 is due to a restricted TCR Vβ family repertoire, as we found a broad spectrum of Vβ families in Art v 1_25-36_–specific cells even within single patients [8]. For DR4-restriction, in general also a broad TCRVβ usage has been reported [12,13] arguing against a lack of specificity for Art v 1_25-36_ in the naïve TCR repertoire. Thus, currently there is no explanation why the DR1-phenotype is highly prevalent in patients who recognize Art v 1_25–36_.

In this study, we investigated the molecular background leading to the preference of HLA-DR1 in the allergic response to the single immunodominant peptide of Art v 1. Potential differences between HLA-DR1 and -DR4 molecules regarding peptide binding and requirements for TCR recognition were addressed. We compared i) presentation of Art v 1_25–36_ by APC expressing either HLA-DR1 or -DR4 to allergen-specific T cell lines (TCL) and clones (TCC) and ii) *in vitro* binding of Art v 1_25–36_ to HLA-DR 1 and -DR4 molecules using mutated peptides; furthermore, iii) we performed computer simulations of Art v 1_25–36_-binding to both HLA-molecules using Molecular Dynamics (MD) simulations.

We found that the majority of Art v 1-specific T cells recognize synthetic Art v 1_25–36_ and variants thereof as well as naturally processed peptides presented by DR1 and DR4 in a similar manner. Differences in minimal epitope recognition suggested structural differences for binding to the MHC peptide binding groove. MD simulation supported this conclusion demonstrating that the peptide is much more flexible at both ends and shows a detached structure at the C-terminus, whereas in DR4 the peptide keeps its extended conformation. Together, the strong bias of HLA-DR1 in the response to the immunodominant epitope of Art v 1 apparently is not primarily due to MHC/peptide binding *per se*, but rather seems to be based on structural differences in the TCR-binding site of the peptide/MHC (pMHC) complex.

## Methods

### Patients

Eleven mugwort pollen-allergic individuals with typical clinical history, i.e. recurrent rhinitis/conjunctivitis during late summer, positive skin prick test (≥3 mm) to mugwort pollen extract (ALK-Abello, Hørsholm, Denmark) and allergen-specific IgE (CAP-FEIA ≥ 3) to mugwort pollen (w6; Phadia, Uppsala, Sweden) were included. All subjects were sensitised to Art v 1 as determined by IgE-immunoblots as described [7]. They expressed the following HLA-DR alleles: Pat.1 DRB1*01, DRB1*11; Pat. 2 DRB1*01, DRB1*15; Pat. 3 DRB1*01, DRB1*13; Pat. 4 DRB1*01, DRB1*03 ; Pat. 5 DRB1*01, DRB1*11; Pat. 6 DRB1*01, DRB1*11; Pat. 7 DRB1*1, DRB1*16; Pat. 8 DRB1*01, DRB1*13; Pat. 9 DRB1*01, DRB1*16, Pat. 10 DRB1*01, DRB1*03; Pat. 11 DRB1*01, DRB1*01; The study was approved by the ethics committee of the Medical University of Vienna (EK No. 497/2005) and informed consent was obtained from all individuals.

### Allergen and peptides

Natural Art v 1 [6] was kindly provided by Matthias Egger (University of Salzburg, Austria). Synthetic peptides were obtained from Thermo Electronics (Ulm, Germany). Purity was >90% as confirmed by HPLC.

### Peptide/MHC-binding assay

HLA-DRB1*01:01 and -DRB1*04:01 molecules were purified from homozygous EBV cells by affinity chromatography using mAb L243 as described before [14]. Peptide binding to HLA-DR molecules was assessed by competitive ELISA. Dilutions were prepared in 10 mM phosphate, 150 mM NaCl,  mM n-dodecyl β-D-maltoside, 10 mM citrate (pH = 6.0). The biotinylated reporter peptide (HA_306-318_) was incubated with HLA-DRB1*01:01 or -DRB1*04:01molecules in the presence of serial dilutions of Art v 1_25-36_ or single mutant peptides thereof. Unlabelled HA_306-318_ peptide was also introduced to assess the validity of each experiment. After 24 hours of incubation at 37°C, peptide/MHC-class II complexes were added to 96 well plates coated with L243 antibody and incubated at RT for 2 hours. Bound biotinylated peptides were detected by Streptavidin-alkaline phosphatase conjugate (GE healthcare, Saclay, France) and 4-methyl-umbelliferyl phosphate (Sigma-Aldrich, St. Quentin Fallavier, France) as substrate. Emitted fluorescence was measured at 450 nm upon excitation at 365 nm by a Gemini fluorometer (Molecular probes, St Grégoire, France). Data are expressed as peptide concentrations that prevented binding of 50% of the labelled reference peptide (IC50) HA_306-318_ (PKYVKQNTLKLAT). Two independent experiments were performed to evaluate competitive binding. Data were reported as relative affinity, i.e. ratio of the IC50 of the peptides to IC50 of the peptide HA_306-318_, to account for the differences of sensitivity of the binding assays.

### Allergen-specific T cell lines (TCL) and T cell clones (TCC)

**Figure 1:**
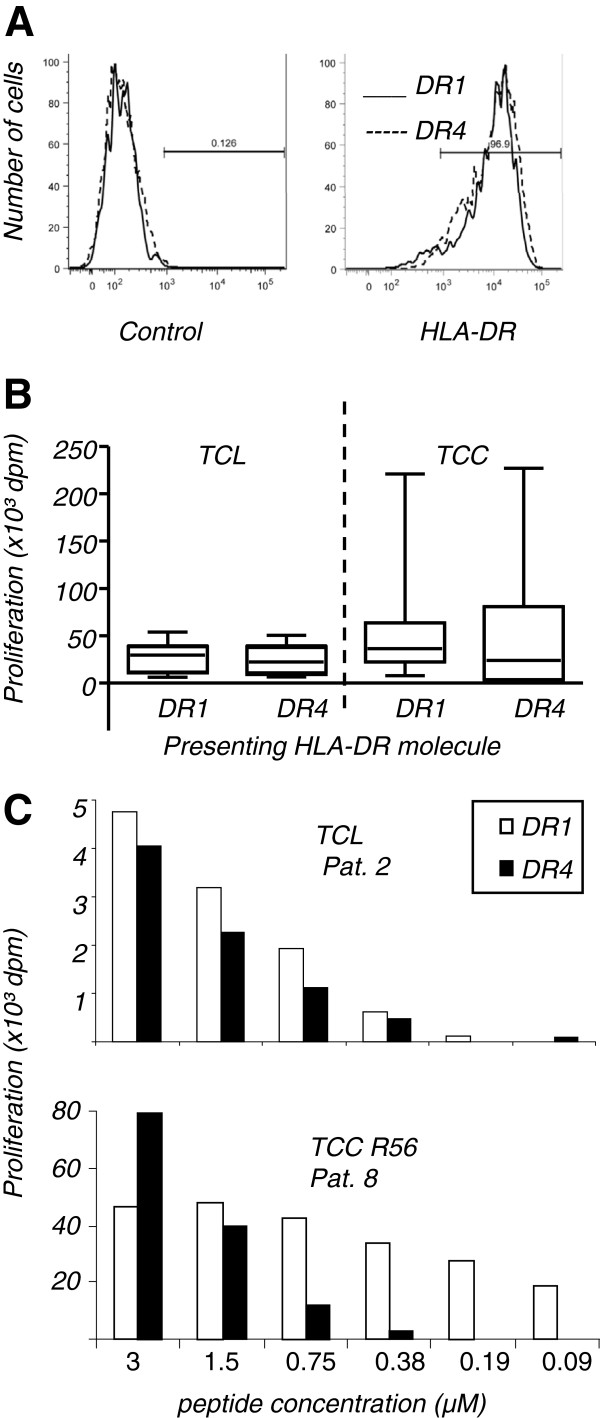
**Presentation of Art v 1**_**25-36**_**by APC expressing HLA-DR1 or -DR4. A.** HLA-DR expression on the EBV cell lines used as APC. **B.** Stimulation of TCL (n = 9) and TCC (n = 17) derived from 5 different subjects with an optimum concentration of Art v 1_25-36_ peptide (3 μM). Box plots indicating the median and quartile ranges are shown. Background proliferations for DR1 or DR4 plus T cells ranged from 1,002-27,612 cpm and 2406-21054 cpm for TCL and from 1525-4264 cpm and 370-4270 cpm for TCC). **C.** T cell reactivity to different concentrations of Art v 1_25-36_ presented by HLA-DR1 or -DR4 T cells were stimulated for 3 days with DR1- or DR4-expressing APC and proliferation was measured by ³H-thymidine uptake during the last 16 hours. Background proliferations were 2796/3056 cpm for Pat 2, 2247/1318 cpm for Pat 8.

Art v 1_25-36_-specific TCL were established from peripheral blood mononuclear cells (PBMC) by allergen-specific stimulation with either mugwort pollen extract or recombinant Art v 1 (Biomay, Vienna, Austria) as previously described [7]. Art v 1_25-36_-specific TCC were established from these TCL by limiting dilution [7]. The two EBV-transformed B cell lines used as APC expressed comparable amounts of HLA-DR molecules (Figure 1A) as well as HLA-DQ, -DP- and the co-stimulatory molecules CD80, CD86, CD40 (not shown) on their surface. TCL or TCC (5 × 10^4^ cells) were stimulated for 3 days with irradiated (60 Gy) APC and peptides at an optimum concentration of 3 μM, if not indicated otherwise. Proliferation was assessed by ^3^H-thymidine uptake within the last 16 hours of incubation. Results are shown in delta cpm (dpm = mean cpm of T cell proliferation with peptide - mean of T cell proliferation without peptide).

### Flow cytometry

EBV-transformed B cell lines were stained with FITC-labelled HLA-DR (L243), DP-(B7/21) and DQ-(SK10) antibodies, CD80 PE, CD86 PE (all BD Biosciences, Franklin Lakes, NJ) and anti CD40 FITC (BioLegend, San Diego, CA) and were analyzed on a FACSCanto with DIVA-software.

### HLA-typing

Typing of HLA-DRB1-alleles was performed using a commercial SSO typing kit (DynalRELI SSO HLA-DRB Typing kit, Dynal, Bromborrough, UK). Samples with only a single detectable DRB1-allele were additionally typed by SSP (Dynal All set SSP DR low resolution, Dynal). High resolution typing was performed by nucleotide sequencing (BigDye Terminator Cycle Sequencing Kit, ABI, Foster City, CA).

### Computer simulation

As starting structure we employed the x-ray structure of HIV GAG(p24)/HLA-DRB1*01:01, Protein Data Bank (PDB) [15] accession code [PDB:1sjh] [16], as it comprises HLA-DR1 in complex with a 13-mer peptide (PEVIPMFSALSEG) which also contains isoleucine as anchor residue similar to Art v 1_25-36_ (KCIEWEKAQHGA). We have previously shown that it is sufficient to mutate the side chains of the peptide and leave its backbone untouched to obtain reliable starting structures [17]. We selected the side-chain substitution tool SCWRL [18] to model Art v 1_25-36_ into the MHC binding groove, since we had found that this tool is the most accurate regarding peptide/MHC interactions [17,19].

As the x-ray structure of HLA-DRB1*04:01 has not been resolved yet and HLA-DRB1*01:01 and HLA-DRB1*04:01 show a sequence identity of 91.58%, we modelled the structure of DRB1*04:01 directly on the basis of the previously created HLA-DRB1*01:01 structure. The differing residues were mutated using SCWRL yielding 2 (pMHC) complexes with identical backbone structure and a few side chain mutations in the MHC.

To perform MD simulations we used the software package GROMACS 4 [20]. First, we immersed both complexes into distinct explicit artificial water boxes. Then we employed a steepest descent energy minimisation of the complexes to resolve spatial clashes and simulated warming to 310 K using position restraints. To provide insight into the dynamics of the pMHC complex at atomic scale, we subsequently carried out MD simulations [21] to investigate spatial rearrangements of molecular structures in water for a real time of 30 ns. All further parameters of the simulation were set according to Omasits *et al*. [22].

We evaluated the resulting trajectories using the root mean square fluctuation (RSMF) which is defined as:

(1) $$\text{RMS}F_{i}=\sqrt{\frac{1}{M}\overset{M}{\underset{k=1}{\sum }}{\left(r_{i}\left(t_{k}\right)-{\tilde{r}}_{i}\right)}^{2}}$$

M is the number of frames, $r_{i}\left(t_{k}\right)$ is particle with number *i* of complex *r* at time *k* and $\tilde{r}$ is the reference structure. This method essentially assesses flexibility of molecules and is implemented in GROMACS in the *g_rmsf* function. Furthermore, we calculated the solvent accessible surface area (SASA) [23] of the side chains of the peptide by rolling a sphere over them [24]. This method is implemented in GROMACS in the *g_sas* function. Visual pre-screening was performed using Visual Molecular Dynamics software (VMD, NIH, University of Illinois) [25] and the plug-in vmdICE [26].

### Statistics

Significance levels were calculated by using Wilcoxon signed rank test (SPSS 10.0, Chicago, IL).

## Results

### Presentation of Art v 1_25-36_ and mutant peptides by HLA-DR1 and -DR4

To compare the peptide presenting capacity of HLA-DR1 and DR4, 9 Art v 1_25-36_-specific TCL from 7 different patients and 17 Art v 1_25-36_-specific TCC derived from 5 different patients were stimulated with the synthetic Art v 1_25-36_ 12-mer peptide in the presence of DR1- or DR4-expressing APC. At optimum peptide concentrations, TCL proliferated slightly less with DR4, although no statistically significant difference was found (Figure 1B; median: 31,630 dpm and 25,985 dpm; p = 0.314). A similar picture was found with the vast majority of TCC with median proliferations of 36,640 dpm to DR1 and 24,125 dpm to DR4 (p = 0.193; Figure 1B). 3/17 (18%) TCC did not respond to presentation by DR4. At lower concentrations of Art v 1_25-36_, APC expressing DR1 outperformed those expressing DR4 (Figure 1C).

**Figure 2:**
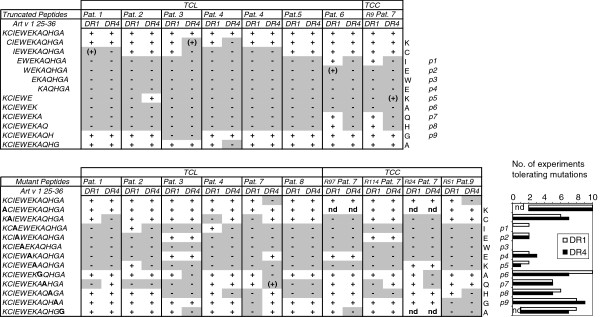
**Mapping of minimal T cell epitopes and critical amino acids within Art v 1**_**25-36**_**presented by HLA-DR1 or -DR4. **T cell reactivity was tested with truncated and single mutant Art v 1_25-36_ –peptides. T cells were stimulated for 3 days with DR1- or DR4-expressing APC and proliferation was measured by ³H-thymidine uptake during the last 16 hours; (+) denotes >50% and (–) >70% reduction of proliferation induced by KCIEWEKAQHGA (dpm). Background proliferations for DR1 or DR4 plus T cells ranged from 1,002-27,612 cpm and 2406-21054 cpm for TCL and from 1525-4264 cpm and 370-4270 cpm for TCC.

Next we determined the minimal T cell epitopes within Art v 1_25-36_ by employing truncated peptides for stimulation of Art v 1_25-36_-reactive TCL (n = 7; 6 different donors) and one TCC (Figure 2A). Minimal epitopes ranged from 5 to 9 aa for DR1 and from 8 to11 aa for DR4. Within these minimal epitopes, the central indispensable aa common in all experiments were EWEKA for DR1 and IEWEKAQH for DR4. In 1 TCC (Pat. 8) and 3 TCL (Pat. 3, 4, 7), the minimal epitope was shorter for DR1 than for DR4 (Figure 2A). For 3/7 Art v 1_25-36_-specific TCL we found identical minimal epitopes for presentation by either DR-molecule.

In general, for peptides which bind to DR1 or DR4, the aa in positions p1 (defined as residue which provides the first anchor), p4, p6 and p9 are supposed to be relevant for HLA-binding, interacting with pockets on the bottom of the binding groove, so that the surface-exposed intermittent aa residues at p2/3, p5, p7/8 can interact with the TCR [11,27]. I27 has previously been identified as anchor residue p1 of Art v 1_25-36_[8]. To identify critical aa residues of Art v 1_25-36_ when presented either by DR1 or DR4, a set of peptide analogues with single aa substitutions was used for T cell stimulations. Besides the crucial anchor residue I27 (p1), W29 (p3) turned out to be indispensable for T cell reactivity in response to presentation by DR1 and DR4 in all TCL (from 6 different donors) and TCC (from 2 donors) tested (Figure 2B). Moreover, E28 (p2), E30 (p4) and K31 (p5) were critical for more than 70% of the T cell responses. The residues C26 (p-1), Q33 (p7), H34 (p8) and A36 (p10) turned out to be additionally important for 30-50% of T cells, again irrespective of presentation by DR1 or DR4 (Figure 2B). A32 (p6) and G35 (p9) represented dispensable residues, possibly due to their small side chains which limit molecular interactions.

Taken together, for the T cell recognition of Art v 1_25-36_ we found indications for a shorter minimal epitope in DR1, but no marked differences in the position and number of residues crucial for interaction with the two HLA-molecules.

### Presentation of naturally-processed Art v 1-peptides by HLA-DR1 and -DR4 molecules

**Figure 3:**
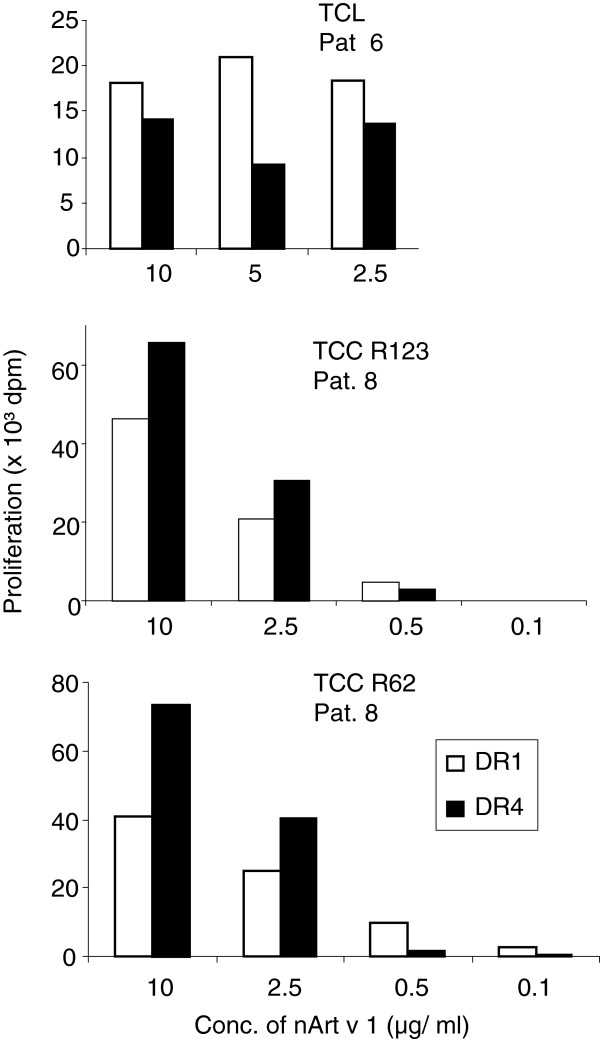
**Presentation of naturally processed Art v 1-peptides by DR1- or DR4-expressing APC to Art v 1**_**25-36**_**-specific TCL and TCC. **T cells were stimulated for 3 days and proliferation was measured by ³H-thymidine uptake during the last 16 hours.

The experiments performed so far had been based on synthetic 12-mer peptides which might bind directly to HLA-DR-molecules expressed on the surface of APC. To compare intracellular loading of processed Art v 1-peptides onto DR1- and DR4-molecules, HLA-DR1- or DR4-expressing APC were incubated with titrated amounts of Art v 1 and used to stimulate Art v 1_25-36_–specific T cell cultures. As observed before with synthetic peptides, the Art v 1_25-36_-specific TCL and 2 TCC proliferated equally well to DR1 or DR4 at optimum concentrations. However, at lower concentrations DR1 tended to be more effective for T cell stimulation (Figure 3).

### Binding of Art v 1_25-36_ to HLA-DR1 and -DR4 molecules

The binding capacity of Art v 1_25-36_ to HLA-DRB1*01:01 and –DRB1*04:01 was assessed in competition studies using HA_306-318_ as reporter peptide (Table 1). HA_306-318_, a high affinity binder to both DR1 and DR4, inhibited itself at an IC50 of 1.8 and 11 nM, respectively. Art v 1_25-36_ was found to bind only 15-fold less to both HLA molecules and hence can still be considered a strong binder for both HLA-molecules. For comparison, we used the neighbouring 12-mer peptides Art v 1_22–33_ and Art v 1_19–30_ which showed weak and negligible binding to DR1 or DR4 molecules, respectively.

**Table 1:** **Binding of Art v 1-peptides and mono-substituted peptides of Art v 1**_**25-36**_to HLA-DRB1*01:01 and HLA-DRB1*04:01

**Peptide**	**Amino Acid sequence**	^**a**^**ratio of IC**_**50**_**of Art v 1-peptides/HA**_**306-318**_		
		**HLA-DR1**	**HLA-DR4**	
HA_304-318_	PKYVKQNTLKLAT	1	1	
Art v _119-30_	NKKCDKKCIEWE	>55032	>926	
Art v 1_22-33_	CDKKCIEWEKAQ	2016	756	
Art v 1_26-36_	KCIEWEKAQHGA	14	15	
Ala-25	**A**CIEWEKAQHGA	9	21	
Ala-26	K**A**IEWEKAGHGA	16	3	
Ala-27	KC**A**EWEKAQHGA	2596	>926	p1
Ala-28	KCI**A**WEKAQHGA	6	11	p2
Ala-29	KCIE**A**EKAQHGA	69	14	p3
Ala-30	KCIEW**A**KAQHGA	6	28	p4
Ala-31	KCIEWE**A**AQHGA	17	17	p5
Gly-32	KCIEWEK**G**QHGA	63	149	p6
Ala-33	KCIEWEKA**A**HGA	11	31	p7
Ala-34	KCIEWEKAQ**A**GA	8	17	p8
Ala-35	KCIEWEKAQH**A**A	5	5	p9
Gly-36	KCIEWEKAQHG**G**	74	242	

^a^ Relative binding activity. Binding of peptides to HLA-DR1 and -DR4 molecules was assessed by competitive inhibition of the high affinity interaction of biotinylated-HA_306-318_ with HLA-DR1 and -DR4 as described in Methods. IC50 values were converted into relative activities (ratio between IC50 value and IC50 of HA_306-318_) to take into account the differences of sensitivity of the binding assays. The IC50 of HA_306-318_ was 1.8 nM for HLA-DR1 and 11 nM for -DR4.

To allocate the aa residues crucial for anchoring the peptide within the groove of the HLA molecule, peptides with single alanine/glycine substitutions at each amino acid position of Art v 1_25–36_ were used. For both HLA-molecules a marked loss of binding was obtained by replacing I27 (Table 1), clearly confirming that this is the anchor residue for HLA-DR1 [8] and indicating that the peptide uses the same anchor residue for binding to DR4. Weaker binding losses for either molecule were induced by glycine substitutions of original alanine residues (A32, A36), which might result from enhancement of the peptide flexibility due to the special properties of glycine. Substitution of W29 resulted in a slight effect on binding to DR1, but not to DR4.

### Computer simulation of Art v 1_25-36_ bound to HLA-DR1 and -DR4

**Figure 4:**
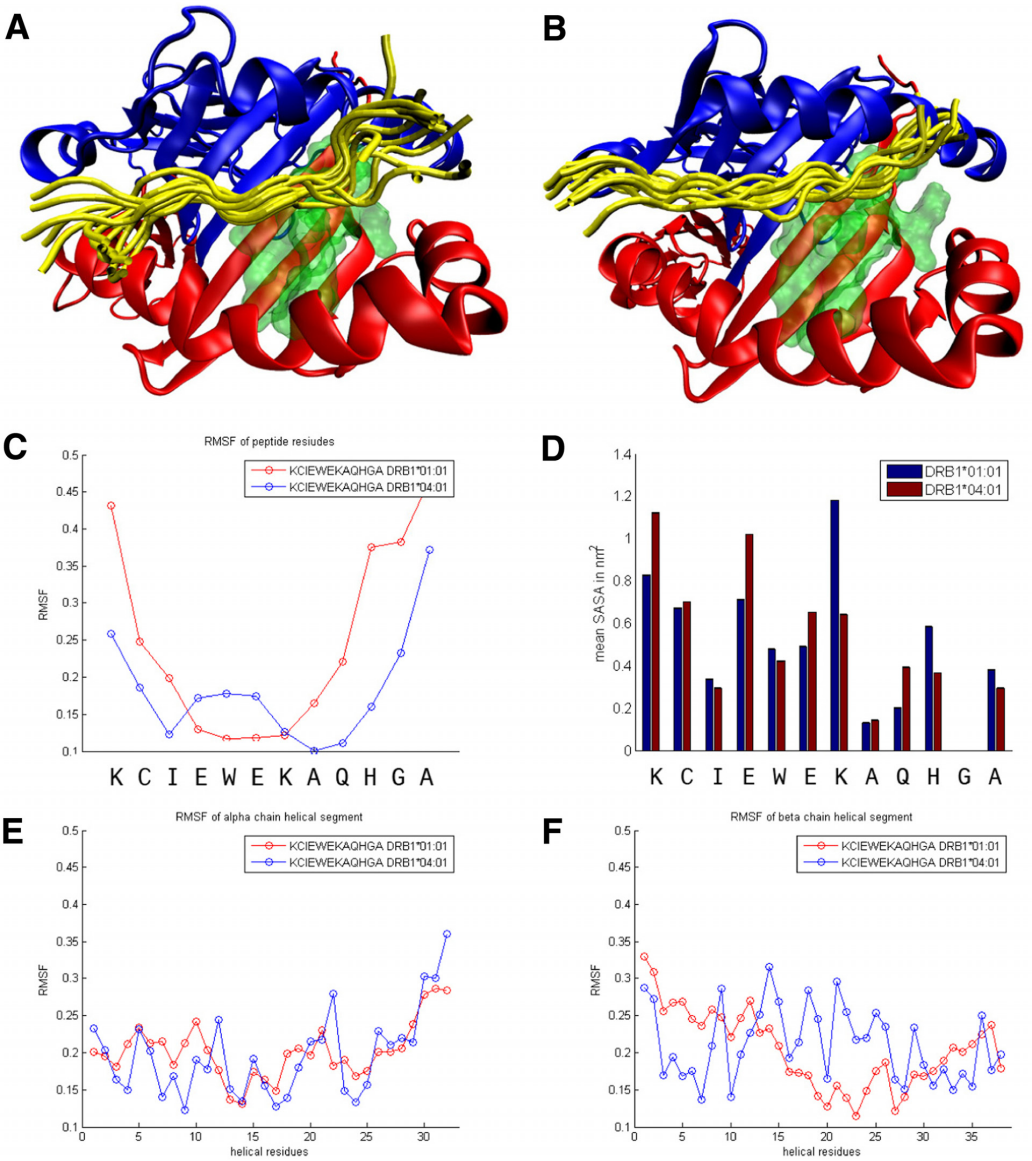
Comparison of the spatial dynamics of HLA-DRB1*01:01 and HLA-DRB1*04:01. (A and B) Cartoon representation of HLA-DRB1*01:01. Blue: MHC alpha chain; Red: MHC beta chain; Yellow: Equally distributed snapshots of the peptide over the whole simulation time; Green: Residues differing between HLA-DRB1*01:01 and HLA-DRB1*04:01 which are within 10 Å of the peptide. **(C)** RMSF of Art v 1_25-36_ bound to HLA-DRB1*01:01 and HLA-DRB1*04:01. **(D)** Difference in average SASA between the two complexes. **(E)** RMSF of the single residues of the helical segment of the MHC alpha chain. **(F)** RMSF of the single residues of the helical segment of the MHC beta chain.

In parallel to our *in vitro* experiments, we investigated structural differences at the level of MHC/peptide binding *in silico* by performing MD simulations. HLA-DRB1*01:01 and HLA-DRB1*04:01 show an aa sequence identity of 91.58%. On this basis the overall tertiary structure is expected to be very similar (see Methods). However, the binding dynamics of Art v 1_25-36_ to each HLA-molecule differed markedly during the simulation time of 30 ns (Figure 4A,B). The interaction with HLA-DR1 was characterized by highly flexible aa residues at the N- (K25) and C-terminus (H34, G35 and A38; Figure 4C) of the peptide with the latter detaching from the binding groove (Figure 4A). In contrast, only A36 showed high flexibility in the interaction with HLA-DR4 (Figure 4C). The different dynamics of Art v 1_25-36_ binding to HLA-DRB1*01:01 and HLA-DRB1*04:01 were also reflected in the mean solvent accessible surface area (SASA) (Figure 4D). K31 and H34 show an increased SASA in HLA-DRB1*01:01 compared to DR4, while the SASA was decreased in the case of K25, E28, E30 and Q33. Interestingly, the increased flexibility of Art v 1_25-36_ in association with HLA-DRB1*01:01 was restricted solely to the peptide itself while the helices of the alpha- and beta-chains of DR1 and DR4 showed a similar behaviour in both molecules (Figure 4E,F). For I27 we found a low flexibility and an almost identical SASA in both complexes indicating that this residue is stably bound to the MHC in both cases.

## Discussion

In spite of the strong association of the allergic immune response to Art v 1 with HLA-DR1, we have previously shown that the *in vitro* binding of its single major T cell epitope Art v 1_25-36_ to HLA-class II molecules and its recognition by CD4+ T cells is degenerate [7,8]. In addition to the vast majority of Art v 1_25-36_-specific, DR1-restricted TCC, a few TCC from DR1-negative patients have been isolated that were restricted by DR15, DR16 or DR3. Although DR4 in general has similar peptide binding characteristics [11] as DR1 and binds Art v 1_25-36_ which can be cross-recognized by DR1-restricted TCC *in vitro*[10], no Art v 1_25-36_–specific DR4-restricted TCC have been isolated so far. Therefore, we expected differences in Art v 1_25-36_–peptide binding and T cell activation between HLA-DR1 and -DR4 and compared anchor residues and minimal epitopes of the peptide to elucidate the molecular background for the DR1-biased T cell response to Art v 1_25-36_ in more detail. To find further support for structural differences between Art v 1_25-36_ bound to HLA-DR1 or HLA-DR4 we also applied MD simulations to the respective pMHC complexes. As there was no crystal structure available for HLA-DR4, our computer simulations on peptide/HLA-DR4 interactions were performed on a modeled structure based on the x-ray structure of HLA-DR1. Nevertheless, this model of HLA-DRB1*04:01 can be assumed to be reliable, as the sequence identity between DR1 and DR4 is above 90% and models based on a template with a sequence identity >50% are generally regarded as highly accurate [28]. Furthermore, major changes in the fold of MHC molecules are extremely unlikely since the MHC superfamily shares a common overall shape [29].

First, we confirmed T cell cross-recognition of Art v 1_25-36_ presented by APC expressing DR4. All T cell cultures used in this study were derived from DR1-positive donors and had most probably been selected by this MHC class II restriction element. Still, the vast majority of TCC (82%) recognized Art v 1_25-36_ presented by DR4 indicating degenerate TCR recognition (Figure 1B). However, at limiting peptide concentrations DR1-expressing APC outperformed DR4-expressing APC in their stimulating capacity (Figure 1C). Natural processing of antigens generates a pool of peptides with different length (mostly 15-18 aa) [30,31] and their intracellular loading into MHC-class II molecules underlies editing by HLA-DM [32]. Similar to our synthetic, 12-mer Art v 1-peptides, the peptides resulting from natural processing were also loaded efficiently into both DR1- and DR4 molecules as detected by the response of Art v 1_25-36_-specific T cells (Figure 3). Here again, DR1-expressing APC performed better at lower peptide concentrations than DR4-expressing APC.

Next, differences in the binding behaviour of Art v 1_25-36_ to DR1- and DR4-molecules and T cell stimulatory capacity were addressed. *In vitro* binding assays with isolated HLA-molecules and single aa mutants of Art v 1_25-36_ confirmed peptide-binding to DR1-molecules with high affinity and residue I27 as p1-anchor [8]. Moreover, we found a similar binding affinity and again I27 as p1-anchor for binding to DR4-molecules (Table 1). The latter was consistent with our computer simulation data. Notably, the residues at p4, p6, or p9 (E30, A32, G35) which in general can bind to additional secondary binding pockets in the groove of DR1 and DR4, had no measurable further supporting effects for Art v 1_25-36_ -binding to either DR molecule, again emphasizing the anchor role of I27. A and G by nature do not provide suitable side chains for anchoring and also proved not to be relevant for T cell activation (Figure 2B). On the other hand, E30 (p4) turned out to be important for most T cell responses (Figure 2). This finding might indicate that, instead of interacting with the HLA-molecule, E30 may interact with the TCR together with its neighbouring aa W29 (p3). However, the calculated surface exposition (SASA) of E30 (p4) obtained by computer modelling was relatively low (Figure 4F) and rather suggested interaction with the MHC molecule. In this case, the simultaneous presence of the all-dominant I27 in the A30-mutant peptide apparently prevented *in vitro* detection of secondary MHC-binding residues.

Overall, the position and number of Art v 1_25-36_-residues crucial for T cell reactivity was similar in response to DR1- or DR4-molecules and focussed on the 5 aa stretch from I27 to K31. The indispensible residues E28 (p2) W29 (p3), K31 (p5) are located in the core of the minimal epitopes found. Less frequently Q33 (p7) and H34 (p8) also formed part of the minimal epitopes (Figure 2A). Due to their increased flexibility and higher surface exposition of H34 in DR1 (Figure 4E,F), these aa may contribute to a more intense TCR-interaction. An influence of peptide flanking residues (p-1 and p10) on T cell responses was also observed in a few experiments, but was not restricted to either DR1- or DR4-presentation.

Although we found similar affinities and structures (i.e. the same register) for Art v 1_25-36_-binding to DR1 and DR4 (Table 1), for more than 50% of the T cells the minimal epitope was shorter for the DR1-bound peptide (Figure 2A). *In silico,* differences in peptide flexibility, indicated by RMSF, became obvious between Art v 1_25-36_ bound to HLA-DR1 or HLA-DR4 with advanced simulation time. In DR1 the peptide was more flexible at both ends and detached from the MHC-molecule at the C-terminus (Figure 4A-C). In essence, the parts of the Art v 1-peptide with lower ranges in RMSF, i. e. less flexibility, reflect the common core of the minimal peptide regions as defined by our *in vitro* experiments for DR1 (IEWEK) or DR4 (CIEWEKAQH). Thus these peptide ranges might define the parts of Art v 1_25-36_ buried in the MHC-binding groove. Further indications for structural differences between the Art v 1_25-36_ /DR1 or -DR4 complex were the overall discrepancies obtained for surface exposition (SASA, Figure 4D). As compared to DR4, the C-terminal aa residues in DR1 were more solvent-exposed, while the N-terminal residues were less exposed. In addition, K31 at position p5, classically the central solvent TCR-exposed residue in MHC class II [33], showed a markedly higher SASA value in complex with DR1 than with DR4. These differences may indicate better access of the TCR to the core and C-terminus of the Art v 1-peptide in DR1.

**Figure 5:**
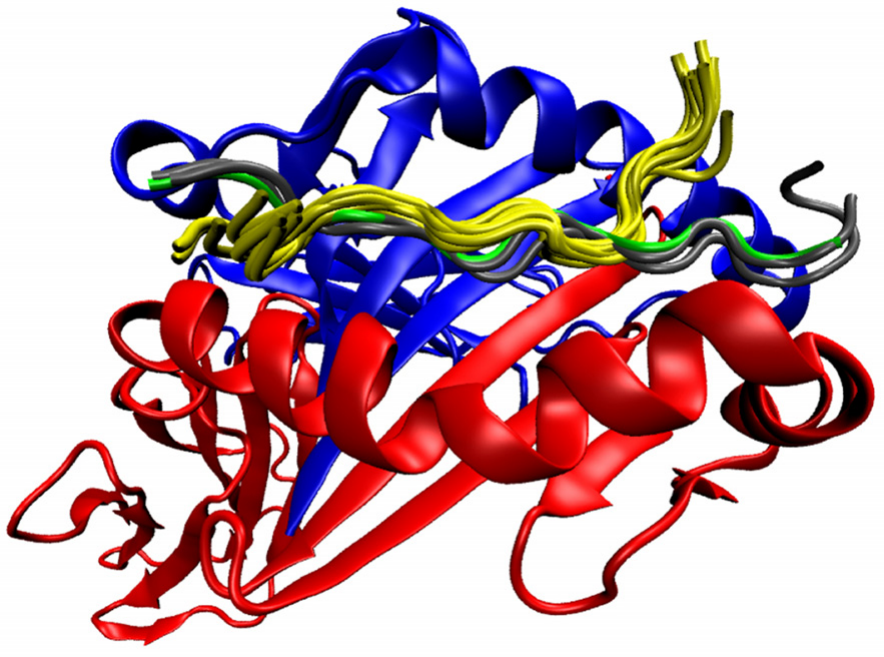
Visualisation of Gag p24 34-48 PEVIPMFSALSEGAT bound to HLA-DRB1*0101 [PDB:1sje] against a sub state of the Art v 1_**25-36**_/HLA-DRB1*01:01 simulation. Blue: MHC alpha chain; Red: MHC beta chain; Yellow: Equally distributed snapshots of Art v 1_25-36_ between nanoseconds 25 and 28; Green: Initial conformation of Art v 1_25-36_ at the beginning of the simulation. Black: peptide conformation of the x-ray structures of [PDB:1sjh] Gag p24 34-46 (13mer peptide) and Gag p24 34-48 [PDB:1sje] (15mer peptide).

Our simulations were based on the crystal structure of the 13-mer peptide HIV Gag p24 _34-46_ PEVIPMFSALSEG bound to HLA-DRB1*0101 [PDB:1sjh]. Interestingly, a congruent crystal structure exists [PDB:1sje] representing HLA-DRB1*01:01 with the 15-mer peptide HIV Gag p24 _34-48_ containing 2 additional C-terminal aa residues (A, T). While the 13 N-terminal residues of the peptide show an essentially identical spatial arrangement as the Art v 1-complex at the initial configuration [16,26], these two residues reach out of the MHC binding groove and build a hairpin turn which has been reported to be essential for T cell recognition of this HIV Gag p24–epitope [16,34]. Intriguingly, this hairpin structure resembles certain sub-states of the detached C-terminal end of the Art v 1_25-36_ / HLA-DRB1*01:01 complex as shown in Figure 5. Within 61 published crystal structures of pMHC class II molecules that contain at least short peptide flanking regions, we found in about 10% of them structures divergent from the canonical stretched conformation such as detachments, loop-like structures or hairpin turns. These structures most likely are important for T cell reactivity. We have previously reported effects of such protruding peptide segments on T cell activation for peptide flanking regions [34] and altered peptide ligands with high sequence similarity [35]. Thus, we suggest that the C-terminal Art v 1_25-36_-residues protruding from the peptide binding groove are relevant for interactions with TCR *in vivo.*

An alternative interpretation for the lack of DR4-responses to Art v 1 in the population may be a potential negative influence of flanking regions in naturally processed peptides selectively on HLA-DR4. However, the fact that DR1-restricted T cells also respond well to naturally processed, DR4-presented Art v 1 (Figure 3) at optimum concentrations indicates that these effects would only be of minor relevance.

In summary, compared to DR4, DR1 allowed T cell activation of shorter minimal epitopes within Art v 1_25-36_ and at lower peptide concentrations. However, the differences obtained in our experimental assays may not clearly explain the high association of Art v 1_25-36_-recognition and HLA-DRB1*01. We used allergen-specific effector T cells with a very high sensitivity for antigen-specific activation in our study. However, the threshold level for activation of naïve T cells is much higher. Therefore, we suggest that at much lower allergen-concentrations, i.e. at conditions present in the human body, the observed differences become much more relevant *in vivo*, so that DR1- but not DR4-expressing APC are capable of activating cognate T cells. This assumption would be backed by the fact that we could not establish DR4-restricted TCC from any of our DR4-expressing mugwort-allergic patients.

## Conclusions

We used the T cell response to the immunodominant epitope of Art v 1 as a model to study p/MHC/TCR interactions in order to explain why a peptide with degenerate MHC-class II binding behaviour causes primarily HLA-DR1-restricted immune responses. The combination of experimental and *in silico* data suggested that TCR recognition of this peptide in the context of DR1 may be promoted by distinctive structural features in the pMHC complex leading e.g. to superior TCR accessibility and preferential priming *in vivo*.

## Abbreviations

Aa: Amino acid; APC: Antigen presenting cell; HLA: Human leukocyte antigen; IC_50_: Concentration causing half-maximum inhibition; MD: Molecular Dynamics; MHC: Major histocompatibility complex; pMHC: Peptide/major histocompatibility complex; PDB: Protein Data Bank; RMSF: Root mean square fluctuation; SASA: Solvent accessible surface area; TCR: T cell receptor; Th: T helper; TCL: T cell line/s; TCC: T cell clone/s.

## Competing interests

BB is consultant for Biomay. All other authors declare that they have no competing interests.

## Authors' contributions

BJS and BK conceived of the study; GF and BB participated in its design and interpretation; BK did the computer simulation studies; BJS and DVH carried out the T cell experiments; IF and GF performed HLA-typing and analysed the data; BM did the HLA-binding assays; WS contributed with materials and supervision; CE provided clinical expertise; BJS and BK prepared the manuscript; BB and GF helped to draft the manuscript. All authors read and approved the final manuscript.

## References

[B1] Romagnani S The role of lymphocytes in allergic disease J Allergy Clin Immunol 2000 105 399 408 10.1067/mai.2000.104575 10719286

[B2] Hammer J Valsasnini P Tolba K Bolin D Higelin J Takacs B Sinigaglia F Promiscuous and allele-specific anchors in HLA-DR-binding peptides Cell 1993 74 197 203 10.1016/0092-8674(93)90306-B 8334703

[B3] Panina-Bordignon P Tan A Termijtelen A Demotz S Corradin G Lanzavecchia A Universally immunogenic T cell epitopes: promiscuous binding to human MHC class II and promiscuous recognition by T cells Eur J Immunol 1989 19 2237 2242 10.1002/eji.1830191209 2481588

[B4] Wilson DB Wilson DH Schroder K Pinilla C Blondelle S Houghten RA Garcia KC Specificity and degeneracy of T cells Mol Immunol 2004 40 1047 1055 10.1016/j.molimm.2003.11.022 15036909

[B5] Ou D Mitchell LA Tingle AJ HLA-DR restrictive supertypes dominate promiscuous T cell recognition: association of multiple HLA-DR molecules with susceptibility to autoimmune diseases J Rheumatol 1997 24 253 261 9034980

[B6] Himly M Jahn-Schmid B Dedic A Kelemen P Wopfner N Altmann F van Ree R Briza P Richter K Ebner C Art v 1, the major allergen of mugwort pollen, is a modular glycoprotein with a defensin-like and a hydroxyproline-rich domain Faseb J 2003 17 106 108 12475905 10.1096/fj.02-0472fje

[B7] Jahn-Schmid B Kelemen P Himly M Bohle B Fischer G Ferreira F Ebner C The T cell response to Art v 1, the major mugwort pollen allergen, is dominated by one epitope J Immunol 2002 169 6005 6011 12421987 10.4049/jimmunol.169.10.6005

[B8] Jahn-Schmid B Fischer GF Bohle B Fae I Gadermaier G Dedic A Ferreira F Ebner C Antigen presentation of the immunodominant T-cell epitope of the major mugwort pollen allergen, Art v 1, is associated with the expression of HLA-DRB1 *01 J Allergy Clin Immunol 2005 115 399 404 10.1016/j.jaci.2004.10.010 15696102

[B9] Jahn-Schmid B Pickl WF Bohle B Interaction of allergens, major histocompatibility complex molecules, and T cell receptors: a 'menage a trois' that opens new avenues for therapeutic intervention in type I allergy Int Arch Allergy Immunol 2011 156 27 42 10.1159/000321904 21447957

[B10] Jahn-Schmid B Sirven P Leb V Pickl WF Fischer GF Gadermaier G Egger M Ebner C Ferreira F Maillere B Characterization of HLA class II/peptide-TCR interactions of the immunodominant T cell epitope in Art v 1, the major mugwort pollen allergen J Immunol 2008 181 3636 3642 18714038 10.4049/jimmunol.181.5.3636

[B11] Southwood S Sidney J Kondo A del Guercio MF Appella E Hoffman S Kubo RT Chesnut RW Grey HM Sette A Several common HLA-DR types share largely overlapping peptide binding repertoires J Immunol 1998 160 3363 3373 9531296

[B12] Grunewald J Shankar N Wigzell H Janson CH An analysis of alpha/beta TCR V gene expression in the human thymus Int Immunol 1991 3 699 702 10.1093/intimm/3.7.699 1832951

[B13] He X Rosloniec EF Myers LK McColgan WL 3rd Gumanovskaya M Kang AH Stuar JM T cell receptors recognizing type II collagen in HLA-DR-transgenic mice characterized by highly restricted V beta usage Arthritis Rheum 2004 50 1996 2004 10.1002/art.20289 15188377

[B14] Texier C Pouvelle S Busson M Herve M Charron D Menez A Maillere B HLA-DR restricted peptide candidates for bee venom immunotherapy J Immunol 2000 164 3177 3184 10706708 10.4049/jimmunol.164.6.3177

[B15] Berman HM Westbrook J Feng Z Gilliland G Bhat TN Weissig H Shindyalov IN Bourne PE The Protein Data Bank Nucleic Acids Res 2000 28 235 242 10.1093/nar/28.1.235 10592235 PMC102472

[B16] Zavala-Ruiz Z Strug I Walker BD Norris PJ Stern LJ A hairpin turn in a class II MHC-bound peptide orients residues outside the binding groove for T cell recognition Proc Natl Acad Sci U S A 2004 101 13279 13284 10.1073/pnas.0403371101 15331779 PMC516560

[B17] Knapp B Omasits U Schreiner W Side chain substitution benchmark for peptide/MHC interaction Protein Sci 2008 17 977 982 10.1110/ps.073402508 18434501 PMC2386744

[B18] Canutescu AA Shelenkov AA Dunbrack RL Jr.: A graph-theory algorithm for rapid protein side-chain prediction Protein Sci 2003 12 2001 2014 10.1110/ps.03154503 12930999 PMC2323997

[B19] Knapp B Omasits U Frantal S Schreiner W A critical cross-validation of high throughput structural binding prediction methods for pMHC J Comput Aided Mol Des 2009 23 301 307 10.1007/s10822-009-9259-2 19194661

[B20] Hess B Kutzner C VanderSpoel D Lindahl E GROMACS 4: Algorithms for Highly Efficient, Load-Balanced, and Scalable Molecular Simulation J Chem Theory Comput 2008 4 435 447 10.1021/ct700301q 26620784

[B21] Karplus M McCammon JA Molecular dynamics simulations of biomolecules Nat Struct Biol 2002 9 646 652 10.1038/nsb0902-646 12198485

[B22] Omasits U Knapp B Neumann M Steinhauser O Stockinger H Kobler R Schreiner W Analysis of Key Parameters for Molecular Dynamics of pMHC Molecules Mol Simulat 2008 34 781 793 10.1080/08927020802256298

[B23] Lee B Richards FM The interpretation of protein structures: estimation of static accessibility J Mol Biol 1971 55 379 400 10.1016/0022-2836(71)90324-X 5551392

[B24] Shrake A Rupley JA Environment and exposure to solvent of protein atoms Lysozyme and insulin. J Mol Biol 1973 79 351 371 10.1016/0022-2836(73)90011-9 4760134

[B25] Humphrey W Dalke A Schulten K VMD: visual molecular dynamics J Mol Graph 1996 14 33 38 27-28 10.1016/0263-7855(96)00018-5 8744570

[B26] Knapp B Lederer N Omasits U Schreiner W vmdICE: a plug-in for rapid evaluation of molecular dynamics simulations using VMD J Comput Chem 2010 31 2868 2873 20928849 10.1002/jcc.21581

[B27] Sette A Sidney J Oseroff C del Guercio MF Southwood S Arrhenius T Powell MF Colon SM Gaeta FC Grey HM HLA DR4w4-binding motifs illustrate the biochemical basis of degeneracy and specificity in peptide-DR interactions J Immunol 1993 151 3163 3170 7690794

[B28] Baker D Sali A Protein structure prediction and structural genomics Science 2001 294 93 96 10.1126/science.1065659 11588250

[B29] Ehrenmann F Kaas Q Lefranc MP IMGT/3Dstructure-DB and IMGT/DomainGap Align: a database and a tool for immunoglobulins or antibodies, T cell receptors, MHC, IgSF and MhcSF Nucleic Acids Res 2010 38 D301 D307 10.1093/nar/gkp946 19900967 PMC2808948

[B30] Chicz RM Urban RG Lane WS Gorga JC Stern LJ Vignali DA Strominger JL Predominant naturally processed peptides bound to HLA-DR1 are derived from MHC-related molecules and are heterogeneous in size Nature 1992 358 764 768 10.1038/358764a0 1380674

[B31] Mutschlechner S Egger M Briza P Wallner M Lackner P Karle A Vogt AB Fischer GF Bohle B Ferreira F Naturally processed T cell-activating peptides of the major birch pollen allergen J Allergy Clin Immunol 2010 125 711 718 718 e1-718 e2 10.1016/j.jaci.2009.10.052 20132976

[B32] Kropshofer H Vogt AB Moldenhauer G Hammer J Blum JS Hammerling GJ Editing of the HLA-DR-peptide repertoire by HLA-DM Embo J 1996 15 6144 6154 8947036 PMC452435

[B33] Reinherz EL Tan K Tang L Kern P Liu J Xiong Y Hussey RE Smolyar A Hare B Zhang R The crystal structure of a T cell receptor in complex with peptide and MHC class II Science 1999 286 1913 1921 10.1126/science.286.5446.1913 10583947

[B34] Knapp B Omasits U Bohle B Maillere B Ebner C Schreiner W Jahn-Schmid B 3-Layer-based analysis of peptide-MHC interaction: in silico prediction, peptide binding affinity and T cell activation in a relevant allergen-specific model Mol Immunol 2009 46 1839 1844 10.1016/j.molimm.2009.01.009 19232439

[B35] Knapp B Omasits U Schreiner W Epstein MM A comparative approach linking molecular dynamics of altered peptide ligands and MHC with in vivo immune responses PLoS One 2010 5 e11653 10.1371/journal.pone.0011653 20657836 PMC2906508

